# Effects of Intragastric Administration of Tryptophan on the Blood Glucose Response to a Nutrient Drink and Energy Intake, in Lean and Obese Men

**DOI:** 10.3390/nu10040463

**Published:** 2018-04-08

**Authors:** Sina S. Ullrich, Penelope C. E. Fitzgerald, Pieter Giesbertz, Robert E. Steinert, Michael Horowitz, Christine Feinle-Bisset

**Affiliations:** 1 Adelaide Medical School and National Health and Medical Research Council of Australia Centre of Research Excellence in Translating Nutritional Science to Good Health, Level 5 Adelaide Health and Medical Sciences Building, Corner North Terrace and George Street, Adelaide 5005, Australia; sina.ullrich@adelaide.edu.au (S.S.U.); penelope.fitzgerald@adelaide.edu.au (P.C.E.F.); re.steinert@gmail.com (R.E.S.); michael.horowitz@adelaide.edu.au (M.H.); 2 Department of Nutritional Physiology, Technical University of Munich, Gregor-Mendel Strasse 2, 85354 Freising, Germany; pieter.giesbertz@tum.de; 3 Department of Surgery, Division of Visceral and Transplantation Surgery, University Hospital Zürich, Rämistrasse 100, 8091 Zürich, Switzerland

**Keywords:** insulin, glucagon, glycemic control, cholecystokinin, humans, food intake

## Abstract

Tryptophan stimulates plasma cholecystokinin and pyloric pressures, both of which slow gastric emptying. Gastric emptying regulates postprandial blood glucose. Tryptophan has been reported to decrease energy intake. We investigated the effects of intragastric tryptophan on the glycaemic response to, and gastric emptying of, a mixed-nutrient drink, and subsequent energy intake. Lean and obese participants (*n* = 16 each) received intragastric infusions of 1.5 g (“Trp-1.5g”) or 3.0 g (“Trp-3.0g”) tryptophan, or control, and 15 min later consumed a mixed-nutrient drink (56 g carbohydrates). Gastric emptying (^13^C-acetate breath-test), blood glucose, plasma C-peptide, glucagon, cholecystokinin and tryptophan concentrations were measured (*t* = 0–60 min). Energy intake was assessed between *t* = 60–90 min. In lean individuals, Trp-3.0g, but not Trp-1.5g, slowed gastric emptying, reduced C-peptide_AUC_ and increased glucagon_AUC_ (all *P* < 0.05), but did not significantly decrease the blood glucose response to the drink, stimulate cholecystokinin or reduce mean energy intake, compared with control. In obese individuals, Trp-3.0g, but not Trp-1.5g, tended to slow gastric emptying (*P* = 0.091), did not affect C-peptide_AUC_, increased glucagon_AUC_ (*P* < 0.001) and lowered blood glucose at *t* = 30 min (*P* < 0.05), and did not affect cholecystokinin or mean energy intake. In obese individuals, intragastrically administered tryptophan may reduce postprandial blood glucose by slowing gastric emptying; the lack of effect on mean energy intake requires further investigation.

## 1. Introduction

The slowing of gastric emptying and the release of gut and pancreatic hormones both play important roles in the regulation of food intake and postprandial blood glucose. For example, cholecystokinin (CCK) decreases energy intake [1,2], and gastric emptying is a major determinant of the initial (~15–30 min) glycemic response to carbohydrate–containing meals in health, obesity and type 2 diabetes [3,4,5,6]. Thus, slowing of gastric emptying attenuates the initial postprandial rises in both blood glucose and insulin [7], and, by prolonging gastric distension, may also increase fullness [8]. The rate of gastric emptying and the magnitude of the release of gut hormones are interdependent processes influenced by the nutrients ingested [9,10,11]. Thus, specific nutrients may potently activate gastrointestinal (GI) functions that are involved in the regulation of blood glucose and/or energy intake.

The aromatic amino acid, tryptophan, is of particular interest as it potently stimulates GI functions [12,13,14,15] and also serves as a precursor for the neurotransmitter serotonin, a key regulator of GI motility and appetite [16]. We have reported that intraduodenal infusion of tryptophan at doses of ~0.41–3.3 g stimulates pyloric pressures, a key regulator of gastric emptying, in lean and overweight volunteers [12,15] and increases plasma CCK and glucagon concentrations [12]. When ingested orally in capsules (dose: ~3 g) 45 min before a lunch meal, or administered intraduodenally over 90 min (dose ~3.3 g), tryptophan also markedly decreased energy intake in healthy subjects [12,17]. In the latter study [12], we established in a pilot trial that while the 3.3 g dose was well tolerated, an intraduodenal dose of 4.4 g was associated with significant adverse effects. The magnitude of energy intake suppression induced by the 3.3 g dose correlated with plasma glucagon and CCK, and most closely correlated with plasma tryptophan concentrations. This is consistent with the concept that the effects of tryptophan on energy intake are mediated by both pre-absorptive (GI) and post-absorptive (including possibly central) effects. Interestingly, the increase in plasma tryptophan concentrations in response to oral tryptophan in doses of 0.25–1 g ingested with a 30 g sucrose drink was found to be ~27% less in obese subjects, compared to lean subjects after ingestion of the 1 g dose [18]; this suggests that tryptophan absorption or metabolism may be altered in obesity. The effects of intragastric tryptophan (as opposed to oral tryptophan, which may influence findings due to its unpleasant taste) on gastric emptying and gut hormone responses to a liquid, mixed-nutrient meal, and on subsequent energy intake, are unknown. It is also uncertain whether slowing of gastric emptying by tryptophan reduces the blood glucose response to oral carbohydrate, and whether these effects are related to plasma tryptophan concentrations. Finally, it remains to be established whether the effects of tryptophan are maintained in the obese, in light of potential changes in tryptophan metabolism in these individuals [18].

The aims of this study were, therefore, to investigate the effects of intragastric tryptophan, at doses of 1.5 g or 3.0 g, based on previous studies [12,14,17], on the blood glucose response to and gastric emptying of a mixed-nutrient drink and subsequent energy intake in lean and obese volunteers. Other parameters relevant to blood glucose and energy intake regulation—specifically plasma C-peptide, glucagon, CCK and tryptophan concentrations—were also quantified. C-peptide was assessed as a more accurate measurement of insulin release, as it is not metabolized by the liver [19]. We hypothesized that (i) tryptophan would lower blood glucose predominantly by slowing gastric emptying, rather than via insulin stimulation; and (ii) the effects of tryptophan on gastric emptying, hormones, blood glucose and energy intake would be maintained in obese volunteers.

## 2. Materials and Methods

Sixteen lean (age: 31 ± 3 years, BMI: 22.1 ± 0.6 kg/m^2^) and 16 obese, non-diabetic subjects (age: 32 ± 3 years, BMI: 33.2 ± 0.6 kg/m^2^, HbA1c 5.3 ± 0.1%) participated in this study. Subjects were screened prior to their inclusion to exclude GI symptoms or surgery, diabetes, low ferritin or iron levels, lactose intolerance, vegetarians, smokers, protein supplement consumption, alcohol consumption of >2 drinks (20 g ethanol) on >5 days a week, high performance athletes or restrained eaters (score > 12 on the restrained eating component of the three factor eating questionnaire [20]). In obese subjects, the latter was not used as an exclusion criterion, as many obese people exhibit some degree of eating restraint [21]. After inclusion, each subject was allocated to a treatment order of balanced randomization [22] by a research officer not involved in data analysis (P.C.E.F.). The study protocol was approved by the Royal Adelaide Hospital Human Research Ethics Committee, and performed in accordance with the Declaration of Helsinki. All subjects provided written informed consent prior to their inclusion. The study was registered as a clinical trial with the Australia and New Zealand Clinical Trials Registry [23] (trial number: 12613000899741).

### 2.1. Study Design

We investigated in normal-weight and obese volunteers the effects of two doses of tryptophan (1.5 g (“Trp-1.5g”) and 3.0 g (“Trp-3.0g”)), or control, administered intragastrically, on gastric emptying of, and the blood glucose and plasma hormone and tryptophan responses to, a mixed-macronutrient drink, given 15 min after the intragastric infusion (*t* = 0 min). For the hour after the drink, blood samples for blood glucose, plasma C-peptide, glucagon, CCK and tryptophan concentrations, ratings of appetite perceptions and GI symptoms were collected every 15 min, and breath samples, for the measurement of gastric emptying, every 5 min. Energy intake from a cold, buffet-style meal was assessed immediately afterwards, i.e., at *t* = 60–90 min. Treatments were administered on three separate occasions in a randomized, double-blind fashion. Study days were separated by at least 2, and up to 7, days.

### 2.2. Intragastric Infusions

A total of 1.5 g or 3.0 g l-tryptophan (PureBulk Inc., Roseburg, OR, USA), 58 mg CaCl_2_xH_2_O, and 1.75 g or 1.65 g NaCl, respectively, were dissolved in 200 mL water for irrigation. Control infusions consisted of 58 mg CaCl_2_xH_2_O and 1.85 g NaCl in 200 mL water. The solutions were approximately isotonic (mOsm, control: 295, Trp-1.5g: 317, Trp-3.0g: 340) and had a pH of ~7 and a temperature of ~23 °C. Solutions were prepared by a research officer (P.C.E.F.) on the morning of each study and infused via a nasogastric catheter directly into the stomach. Syringes were covered to blind both study participants and the investigator performing the study (S.S.U.).

### 2.3. Protocol

Participants were provided with a standardized meal (Beef Lasagna, McCain Food, Wendouree, Victoria, Australia; energy content: 591 kcal), to be consumed between 6:30 p.m. and 7:00 p.m. on the night prior to each study, and instructed to refrain from strenuous exercise and alcohol for 24 h before each study, and from solids and liquids, except water, after the evening meal until they arrived in the laboratory at the Royal Adelaide Hospital at 9:30 a.m. the next morning. Upon arrival, an intravenous cannula was placed into a forearm vein for regular blood sampling. At *t* = −20 min (~9:40 a.m.), which was defined as ‘baseline’, a blood sample for blood glucose and plasma hormone and tryptophan measurements, and a baseline breath sample, as a reference prior to the measurement of gastric emptying, were taken. The subject also completed a visual analog scale (VAS) questionnaire to assess appetite-related perceptions and GI symptoms. Participants were then seated in an upright position and intubated with a soft-silicon feeding tube (outer diameter: 4 mm; Dentsleeve, Mississauga, Ontario, Canada), which was inserted through an anesthetized nostril into the stomach. Immediately thereafter (*t* = −18 min), subjects received the 200-mL intragastric infusion of control or either dose of tryptophan within 2 min. The tube was then removed and 15 min later (*t* = −1 min), subjects completed a VAS questionnaire and consumed, within 1 min, 300 mL of a mixed-nutrient drink (Ensure plus^®^; Abbott, Macquarie Park, New South Wales, Australia; 400 kcal, 56 g carbohydrates, including corn syrup, maltodextrin and sucrose, 15 g protein and 12 g fat) labelled with 100 mg of ^13^C-acetate for measurement of gastric emptying by breath test [24]. Immediately after the drink (*t* = 0 min), and for the following hour (*t* = 0–60 min), breath samples were collected every 5 min, and VAS ratings and blood samples every 15 min. At *t* = 60 min (~11:10 a.m.), participants were presented with a standardized, cold, buffet-style test-meal, described previously, and instructed to eat until they were comfortably full, for up to 30 min (*t* = 60–90 min) [25]. The meal included food in excess of what participants were expected to consume and had a total energy content of 2822 kcal [25]. Participants were unaware that the purpose of the buffet-meal was to assess energy intake. After the buffet-meal (*t* = 90 min), the cannula was removed and participants were free to leave the laboratory.

### 2.4. Measurements

#### 2.4.1. Blood Glucose and Plasma Hormone Analysis

Blood samples were collected into ice-chilled ethylenediaminetetraacetic acid-containing tubes. Plasma was obtained by centrifugation at ~1832 g-force for 15 min at 4 °C within 15 min of collection and stored at –80 °C until subsequent analysis.

Blood glucose concentrations (millimoles per liter) were determined immediately after sampling using a portable glucometer (FreeStyle OptiumH, Abbott Laboratories, Doncaster, Victoria, Australia).

Plasma C-peptide concentrations (nanomoles per liter) were measured by enzyme-linked immunosorbent assay (10-1136-01, Mercodia, Uppsala, Sweden). The sensitivity of the assay was 0.015 nmol/L, and intra- and inter-assay coefficients of variation (CVs) were 3.7% and 7.4% respectively.

Plasma glucagon concentrations (picograms per milliliter) were measured by radioimmunoassay (GL-32K, Millipore, Billerica, Massachusetts, USA). The sensitivity of the assay was 20 pg/mL, and intra- and inter-assay CVs were 3.1% and 11.7% respectively.

Plasma CCK-8 concentrations (picomoles per liter) were measured by radioimmunoassay using an adaptation of the method of Santangelo et al. [26]. The sensitivity of the assay was 1 pmol/L, and intra- and inter-assay CVs were 5.3% and 10.9%, respectively.

Plasma tryptophan concentrations (micromoles per liter) were analyzed using targeted LC-MS/MS based on the method described by Harder et al. [27]. Briefly, plasma samples (10 µL) were dissolved in 500 µL ice-cold methanol containing d5-tryptophan (Sigma-Aldrich, Taufkirchen, Germany) as an internal standard. After centrifugation (10 min, 10 °C), supernatant was collected and dried. Amino acids were derivatized to their butyl esters as described by Gucciardi et al. [28]. In short, a mixture of 95% n-butanol and 5% acetylchloride (*v*/*v*) was added to the dried samples. The samples were incubated at 60 °C for 15 min at 600 rpm (Eppendorf Thermomixer Comfort; Eppendorf, Hamburg, Germany), then dried and reconstituted in a 200-µL mixture of methanol/water/formic acid (70/30/0.1% *v*/*v*). The analysis was performed on a triple quadrupole QTRAP 5500 LC-MS/MS system operating in positive electrospray ionization mode (AB Sciex, Framingham, MA, USA) equipped with a 1200 series binary pump (Agilent, Santa Clara, CA, USA) coupled to an HTC pal autosampler (CTC Analytics, Zwingen, Switzerland). Chromatographic separation was achieved using a Zorbax Eclipse XDB-C18 column (length: 150 mm, internal diameter: 3.0 mm, particle size: 3.5 µm; Agilent). The measurement was performed using scheduled multiple reaction monitoring. For absolute quantification, a 10-point calibration of tryptophan concentrations between 1 µM and 500 µM was generated. Data were analyzed using Analyst 1.5.1^®^ software (AB Sciex, Cambridge, UK).

#### 2.4.2. Gastric Emptying

^13^CO_2_ concentrations in end-expiratory breath samples were analyzed using an Automated Breath ^13^Carbon Isotope Ratio Mass Spectrometer (ABCA IRMS, Sercon, UK). Data were expressed as % recovery of ^13^CO_2_ in the breath per hour.

#### 2.4.3. Appetite Perceptions, GI Symptoms and Food Intake

Perceptions of hunger, fullness, desire-to-eat and prospective food consumption were measured using validated 100-mm VAS questionnaires [29]. Nausea and bloating were also assessed. The amount of food consumed was quantified by weighing each food item of the buffet-meal before and after it was presented to the participant. Energy intake (kcal) was calculated using commercially available software (Foodworks 8.0, Xyris Software, Highgate Hill, Queensland, Australia).

### 2.5. Data and Statistical Analyses

The number of subjects studied was determined by power calculations based on our previous work [12,30]. We calculated that *n* = 11 subjects would allow detection of a 1.0 mmol/L reduction in blood glucose, and *n* = 16 subjects a 170 kcal difference in energy intake, both at α = 0.05, with a power of 80%.

Blood glucose, plasma hormone, tryptophan and gastric emptying data were expressed as raw data. VAS data were expressed as changes from baseline (i.e., *t* = −20 min), to account for variations in baseline values. To assess the effects of tryptophan alone, changes from baseline were calculated at *t* = 0 min for blood glucose, plasma hormone and tryptophan concentrations. To assess the response to the mixed-nutrient drink, blood glucose, plasma hormone, tryptophan, gastric emptying and VAS data were expressed as areas under the curve, calculated from *t* = 0–60 min (AUC_0–60min_) using the trapezoidal rule. Blood glucose and C-peptide concentrations at *t* = 30 min were analyzed to evaluate the contribution of gastric emptying to these outcomes [5,31]. To assess differences between obese and lean participants, the differences in responses to Trp-3.0g, relative to control, were calculated for AUCs_0–60min_ of blood glucose, plasma hormone, tryptophan concentrations and gastric emptying, as well as energy intake. Incremental AUCs_0–60min_ (iAUC_0–60min_) were calculated to assess whether differences (if any) in AUCs between obese and lean participants were due to differences at *t* = 0 min or the result of differences in the magnitude of the change.

Statistical analysis was performed using SPSS software (version 24, IBM, Chicago, Illinois). Data, including changes from baseline at *t* = 0 min, AUCs_0–60min_ for blood glucose, plasma hormones and tryptophan, and blood glucose and C-peptide concentrations at *t* = 30 min, and energy intake were analyzed using repeated-measures analysis of variance (ANOVA), with treatment as a within-subjects factor. Differences between treatments were assessed using paired *t*-tests, with adjustments within each model made using Bonferroni’s correction (i.e., α = 0.05/3). Differences in outcomes between obese and lean subjects were assessed using independent *t*-tests. Pearson’s correlations, using the changes from control for both treatments, were performed to evaluate the relationships between energy intake with AUCs_0–60min_ or iAUCs_0–60min_ of plasma tryptophan, hormones, blood glucose and gastric emptying. Differences were considered statistically significant at *P* ≤ 0.05. All data are reported as means ± SEM.

## 3. Results

All participants completed the study and tolerated the study conditions well; three participants (one lean and two obese) felt light-headed, and one (lean) reported mild nausea after Trp-3.0g. In all cases, these symptoms were transient and resolved within ~5 min.

### 3.1. Normal-Weight Subjects

#### 3.1.1. Blood Glucose

**Figure 1:**
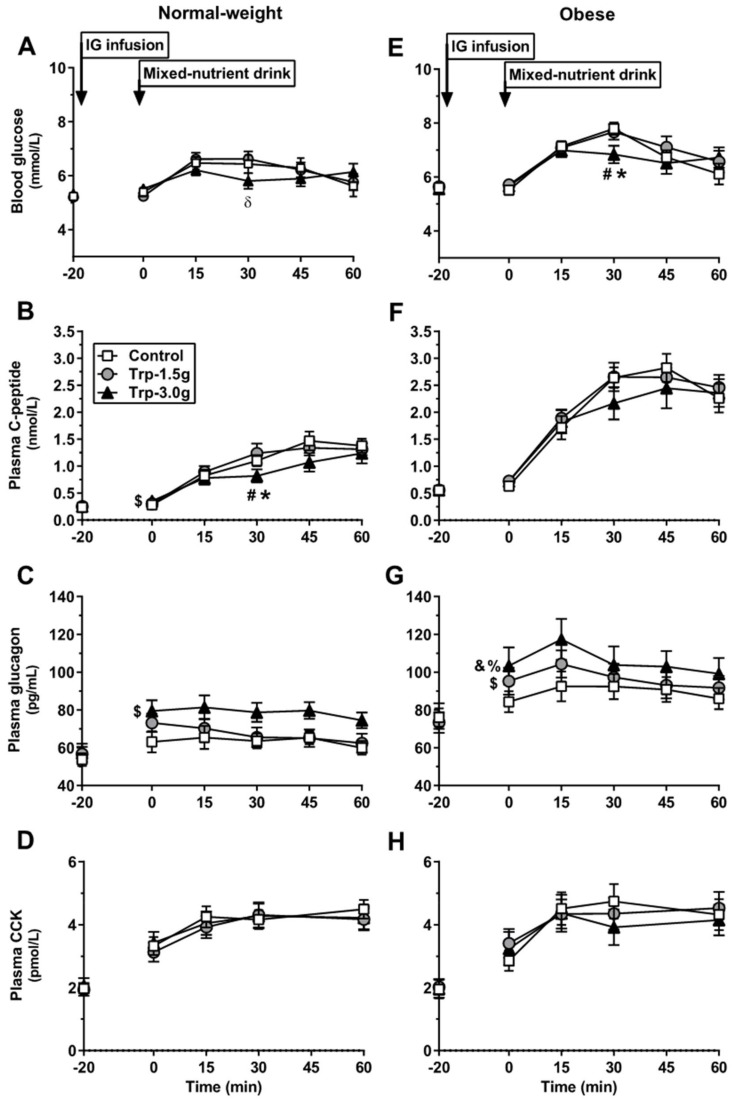
Blood glucose (**A**,**E**), plasma C-peptide (**B**,**F**), glucagon (**C**,**G**) and cholecystokinin (CCK) (**D**,**H**) concentrations at baseline (*t* = −20 min) and in response to an oral mixed-nutrient drink (*t* = 0 min) following intragastric (IG) infusion of tryptophan, at doses of 1.5 g (‘Trp-1.5g’) or 3.0 g (‘Trp-3.0g’), or control, in healthy normal-weight (A–D) and obese (E–H) volunteers. Data are means ± SEM, *n* = 16. Changes from baseline at *t* = 0 min, and blood glucose and C-peptide concentrations at *t* = 30 min, were analyzed using repeated-measures ANOVA. Differences between treatments were assessed using paired *t*-tests, with adjustments for multiple comparisons made within each outcome using Bonferroni’s correction. (**A**) Blood glucose at *t* = 30 min was lower after Trp-3.0g compared with Trp-1.5g (δ *P* < 0.01); (**B**) the change from baseline at *t* = 0 min was greater with Trp-3.0g compared with control ($ *P* < 0.05); C-peptide at *t* = 30 min was lower after Trp-3.0g compared with (*) control and (#) Trp-1.5g (*P* < 0.05 for both); (**C**) the increase from baseline at *t* = 0 min was greater after Trp-3.0g compared with control ($ *P* < 0.05); (**E**) blood glucose at *t* = 30 min was lower after Trp-3.0g compared with (*) control and (#) Trp-1.5g (*P* < 0.05 for both); (**G**) the increase from baseline at *t* = 0 min was greater after Trp-3.0g compared with control (% *P* < 0.001) and Trp-1.5g (& *P* < 0.05), and after Trp-1.5g compared with control ($ *P* < 0.05).

Response to tryptophan: At *t* = 0 min, blood glucose remained at fasting concentrations, with no differences between treatments (Figure 1A).

Response to mixed-nutrient drink: Blood glucose increased slightly on each day, with no differences in AUCs_0–60min_ between treatments (Table 1). Blood glucose at *t* = 30 min was, however, lower after Trp-3.0g compared with Trp-1.5g (*P* < 0.01), but did not reach significance when compared with control (*P* = 0.057), with no difference between Trp-1.5g and control.

#### 3.1.2. C-Peptide

Response to tryptophan: At *t* = 0 min, the increase from baseline was slightly greater after Trp-3.0g compared with control (*P* < 0.05), with no differences between Trp-3.0g and Trp-1.5g, or between Trp-1.5g and control (Figure 1B).

Response to mixed-nutrient drink: Plasma C-peptide increased gradually on each day; C-peptide AUC_0–60min_ was lower after Trp-3.0g compared with control (*P* < 0.05) and Trp-1.5g (*P* < 0.01), with no difference between Trp-1.5g and control. At *t* = 30 min, C-peptide was lower after Trp-3.0g, compared with control and Trp-1.5g (*P* < 0.05 for both), with no difference between Trp-1.5g and control.

#### 3.1.3. Glucagon

Response to tryptophan: At *t* = 0 min, the increase from baseline was greater after Trp-3.0g, compared with control (*P* < 0.05), with no differences between Trp-1.5g and control or Trp-3.0g (Figure 1C).

Response to mixed-nutrient drink: Plasma glucagon plateaued, and glucagon AUC_0–60min_ remained higher after Trp-3.0g, compared with control and Trp-1.5g (*P* < 0.001 for both; Table 1), with no difference between Trp-1.5g and control.

#### 3.1.4. CCK

Response to tryptophan: At *t* = 0 min, plasma CCK was increased on all study days, with no differences in the change from baseline between treatments (Figure 1D).

Response to mixed-nutrient drink: Plasma CCK increased slightly on each day, without any differences between treatments (Table 1).

#### 3.1.5. Gastric Emptying

**Figure 2:**
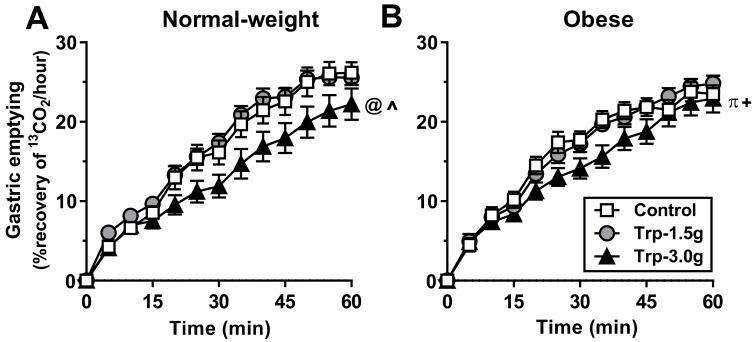
Gastric emptying of a mixed-nutrient drink, ingested at *t* = 0 min, 15 min after an intragastric infusion of tryptophan, at doses of 1.5 g (‘Trp-1.5g’) or 3.0 g (‘Trp-3.0g’), or control, in healthy normal-weight (**A**) and obese (**B**) volunteers. Data are means ± SEM, *n* = 16. Data were analyzed using repeated-measures ANOVA. Differences between treatments were assessed using paired *t*-tests, with adjustments for multiple comparisons made using Bonferroni’s correction. (**A**) Gastric emptying was significantly slower after Trp-3.0g compared with control (^ *P* < 0.05) and Trp-1.5g (@ *P* < 0.001); (**B**) gastric emptying tended to be slower after Trp-3.0g compared with control (+ *P* = 0.091), and was slower after Trp-3.0g compared with Trp-1.5g (π *P* < 0.05).

Gastric emptying of the mixed-nutrient drink was linear and slower after Trp-3.0g compared with control (*P* < 0.05) and Trp-1.5g (*P* < 0.001), with no difference between Trp-1.5g and the control (Figure 2A; Table 1).

#### 3.1.6. Plasma Tryptophan

**Figure 3:**
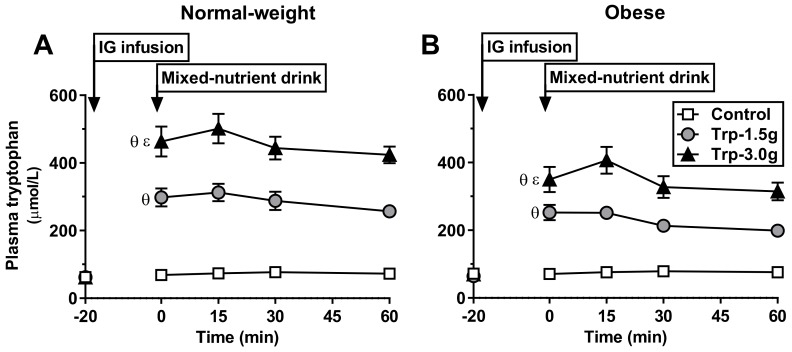
Plasma tryptophan concentrations at baseline (*t* = −20 min) and in response to an oral mixed-nutrient drink (*t* = 0 min) following intragastric (IG) infusion of tryptophan, at doses of 1.5 g (‘Trp-1.5g’) or 3.0 g (‘Trp-3.0g’), or control, in healthy normal-weight (**A**) and obese (**B**) volunteers. Data are means ± SEM, *n* = 16. Changes from baseline at *t* = 0 min, and blood glucose and C-peptide concentrations at *t* = 30 min, were analyzed using repeated-measures ANOVA. Differences between treatments were assessed using paired *t*-tests, with adjustments for multiple comparisons made using Bonferroni’s correction. (**A**) At *t* = 0 min, the increase from baseline was markedly greater after Trp-3.0g compared with (θ) control and (ε) Trp-1.5g, and after Trp-1.5g compared with (θ) control (*P* < 0.001 for all); (**B**) at *t* = 0 min, the increase from baseline was greater after Trp-3.0g, compared with (θ) control and (ε) Trp-1.5g, and after Trp-1.5g compared with (θ) control (*P* < 0.001 for all).

Response to tryptophan: At *t* = 0 min, plasma tryptophan was markedly increased after both tryptophan doses; the change from baseline was greater after Trp-3.0g, compared with control and Trp-1.5g, and greater after Trp-1.5 compared with control (*P* < 0.001 for all; Figure 3A).

Response to mixed-nutrient drink: Plasma tryptophan plateaued on each day and tryptophan AUC_0–60min_ was greater after Trp-3.0g, compared with control and Trp-1.5g, and greater after Trp-1.5g compared with control (*P* < 0.001 for all; Table 1).

#### 3.1.7. Appetite Perceptions, GI Symptoms and Food Intake

Tryptophan did not affect hunger, fullness, desire-to-eat, prospective food consumption (Figure S1), nausea or bloating (Figure S2). While there were no overall differences in energy intake between treatments (kcal, control: 1005 ± 96, Trp-1.5g: 980 ± 108, Trp-3.0g: 907 ± 107), a reduction in energy intake after Trp-3.0g was observed in 8/16 participants (change from control: −311 ± 29 kcal, −35 ± 8%).

### 3.2. Obese Subjects

#### 3.2.1. Blood Glucose

Response to tryptophan: At *t* = 0 min, blood glucose remained at fasting concentrations, with no differences between treatments (Figure 1E).

Response to mixed-nutrient drink: Blood glucose increased on each day, with no differences in AUCs_0–60min_ between treatments (Table 1). However, blood glucose at *t* = 30 min was lower after Trp-3.0g, compared with control and Trp-1.5g (*P* < 0.05 for both), with no difference between Trp-1.5g and control.

#### 3.2.2. C-Peptide

Response to tryptophan: At *t* = 0 min, C-peptide remained at fasting concentrations, with no differences between treatments (Figure 1F).

Response to mixed-nutrient drink: C-peptide increased markedly on each day; however, AUCs_0–60min_, or values at *t* = 30 min, did not differ between treatments (Table 1).

#### 3.2.3. Glucagon

Response to tryptophan: At *t* = 0 min, the increase from baseline was greater after Trp-3.0g, compared with control (*P* < 0.001) and Trp-1.5g (*P* < 0.05), and after Trp-1.5g compared with control (*P* < 0.05; Figure 1G).

Response to mixed-nutrient drink: Glucagon AUC_0–60min_ was higher after Trp-3.0g, compared with control and Trp-1.5g (*P* < 0.01 for both; Table 1), with no difference between Trp-1.5g and control.

#### 3.2.4. CCK

Response to tryptophan: At *t* = 0 min, plasma CCK was increased on all study days, with no differences in the change from baseline between treatments (Figure 1H).

Response to mixed-nutrient drink: Plasma CCK increased on each day, but without any differences between treatments (Table 1).

#### 3.2.5. Gastric Emptying

There was a trend for gastric emptying of the mixed-nutrient drink to be slower after Trp-3.0g compared with control (*P* = 0.091; Figure 2B; Table 1), and gastric emptying was slower after Trp-3.0g compared with Trp-1.5g (*P* < 0.05), with no difference between Trp-1.5g and control.

#### 3.2.6. Plasma Tryptophan

Response to tryptophan: At *t* = 0 min, plasma tryptophan was markedly increased after both tryptophan doses; the increase from baseline was greater after Trp-3.0g compared with control and Trp-1.5g, and greater after Trp-1.5g compared with control (*P* < 0.001 for all; Figure 3B).

Response to mixed-nutrient drink: Plasma tryptophan plateaued on each day and tryptophan AUC_0–60min_ was greater after Trp-3.0g compared with control and Trp-1.5g, and greater after Trp-1.5g compared with control (*P* < 0.001 for all; Table 1).

#### 3.2.7. Appetite Perceptions, GI Symptoms and Food Intake

Tryptophan did not affect hunger, fullness, desire-to-eat, prospective food consumption (Figure S1), nausea or bloating (Figure S2). While there were no overall differences in energy intake between treatments (kcal, control: 1002 ± 55, Trp-1.5g: 984 ± 61, Trp-3.0g: 954 ± 59), a reduction in energy intake was observed after Trp-3.0g in 9/16 participants (change from control: −180 ± 52 kcal; −16.7 ± 4%).

### 3.3. Comparison between Obese and Normal-Weight Subject

After control, AUCs_0–60min_ of blood glucose (*P* < 0.05), plasma C-peptide (*P* < 0.001) and glucagon (*P* < 0.01) were greater in the obese than in the lean. There were no differences in AUCs_0–60min_ of gastric emptying, plasma CCK and tryptophan, or energy intake between obese and lean.

There were no differences in the changes (Trp-3.0g vs. control) in blood glucose, plasma C-peptide, glucagon, CCK, gastric emptying, or energy intake, between obese and lean participants. However, the increase in plasma tryptophan AUC_0–60min_ was less in the obese than the lean (*P* < 0.05). There was no significant difference in the reduction in energy intake in the subjects whose energy intake was reduced after Trp-3g between obese and lean participants.

### 3.4. Relationships among Energy Intake and Blood Glucose with Gastric Emptying, Hormones and Tryptophan Concentrations

There was an inverse correlation between energy intake with plasma tryptophan AUC_0–60min_ (*r* = −0.463, *P* = 0.010) and plasma glucagon AUC_0–60min_ (*r* = −0.450, *P* < 0.05) in lean, but not in obese, subjects. There was a trend for a direct correlation between gastric emptying AUC_0–60min_ with blood glucose iAUC_0–60min_ (*r* = 0.326, *P* = 0.074) in obese subjects. There were inverse correlations between plasma tryptophan AUC_0–60min_ with gastric emptying AUC_0–60min_ in both lean (*r* = −0.536, *P* < 0.01) and obese (*r* = −0.471, *P* = 0.010) subjects.

## 4. Discussion

We have shown that intragastric administration of tryptophan, at a dose of 3 g, decreased the early blood glucose-response to a mixed-nutrient drink in obese, but not significantly in lean, subjects, while slowing gastric emptying in both groups. The rise in C-peptide was attenuated in lean, but not altered in obese, subjects, while glucagon was increased in both groups. Thus, blood glucose was probably lowered primarily as a result of slowed gastric emptying, i.e., slowing of gastric emptying outweighed any potential insulinotropic effect, while the stimulation of glucagon may have prevented a more pronounced decreases in blood glucose. Neither energy intake-suppressant nor CCK-stimulatory effects of tryptophan were observed, in contrast to previous studies, although tryptophan, at 3 g, decreased energy intake in approximately half of the subjects in both groups. Intragastric administration of tryptophan led to a marked, dose-related, elevation of plasma tryptophan, which was greater in the lean than the obese, and energy intake was related to plasma tryptophan concentrations in the lean, but not the obese.

A few studies in both humans and experimental animals have reported that gastric emptying of tryptophan, administered intragastrically or orally, is slowed compared with control, and in a dose-dependent fashion [13,14,32]. Moreover, we have shown that tryptophan, administered intraduodenally, potently stimulates pyloric pressures [15,16]. Our study established that tryptophan, at a dose of 3 g, slows gastric emptying of a mixed-nutrient drink, in normal-weight and, to a lesser extent, in obese volunteers. Our observation that gastric emptying of the drink was not slowed by the lower load of tryptophan suggests that a threshold dose of tryptophan in the small intestine is required to cause sufficient activation of mucosal receptors [33]. The mixed-nutrient drink predictably resulted in only a modest increase in blood glucose in both groups, thus, the magnitude of any decrease in blood glucose induced by tryptophan in our study could only be small. We chose a mixed-nutrient drink over pure glucose, or a high-carbohydrate meal, because it represents a more physiological meal composition. That corn syrup, which contains up to 50% of fructose, was the main carbohydrate in the mixed-nutrient drink, is likely to have contributed to the only modest increase in blood glucose on the control day. In the obese, the postprandial rise in blood glucose was predictably slightly greater, and tryptophan, at 3 g, reduced blood glucose at *t* = 30 min, indicating that this was due to slowed gastric emptying. Our study, therefore, supports previous findings that ingestion of nutrients, which stimulate those aspects of GI function involved in blood glucose regulation, have the capacity to lower postprandial blood glucose responses to carbohydrate-containing meals (the so-called “preload concept”) [10,34], and demonstrates that this can be achieved with much smaller amounts of selected nutrients–in this case, tryptophan.

Tryptophan, infused intraduodenally or intravenously, has been reported to stimulate insulin release [12,35]. In contrast, we found that tryptophan was associated with a reduced insulin response, assessed by measurement of plasma C-peptide, to the mixed-nutrient drink, in the lean, but not the obese. Thus, while tryptophan may have a modest insulin-stimulatory effect in the fasting state, i.e., in absence of other nutrients [12,35], the slower gastric emptying of the mixed-nutrient drink induced by tryptophan led to a diminished, or absent, insulin response, also indicating that the attenuation in postprandial blood glucose was not mediated by insulin. This is now recognized to be the case with postprandial glucose-lowering induced by intravenous GLP-1 [36] and the ‘short-acting’ GLP-1 agonists, exenatide [37] and lixisenatide [38]. It should be appreciated that the blood glucose-lowering effect of tryptophan would almost certainly be greater in patients with type 2 diabetes, in whom pre- and postprandial blood glucose would be substantially greater than observed in our study. Our data extended previous findings indicating that amino acids lower the blood glucose response to the same mixed-nutrient drink via different mechanisms; e.g., while leucine stimulated insulin, isoleucine slowed gastric emptying, and lysine did not affect either [39,40].

Tryptophan has been reported to stimulate glucagon, when infused intraduodenally (overall load: ~3 g), or ingested orally at a dose of 10 g, in the absence of other nutrients, in healthy humans [12,41]. We found a potent, dose-related, stimulation of glucagon that was sustained over the hour after the mixed-nutrient drink in both groups, which would intuitively counteract glucose lowering by increasing hepatic glucose output, preventing a further decrease in blood glucose.

Since previous studies have reported an increase in plasma CCK within ~15 min of intragastric or intraduodenal tryptophan administration, in the absence of other nutrients, in healthy volunteers [12,13], it is surprising that tryptophan did not stimulate plasma CCK in our current study. Based on our previous study, we would have expected an increase in plasma CCK at *t* = 0 min. It should, however, be appreciated that this time-point was after ingestion of the mixed-nutrient drink, and it would not be surprising if some nutrients had already emptied into the small intestine. Accordingly, the capacity to identify any effect of tryptophan on CCK, particularly if it was small, may have been confounded by a CCK-secretory effect of the drink. Thus, it appears that in our study paradigm, CCK does not mediate the effect of tryptophan on gastric emptying, but may relate to a direct effect of tryptophan on small intestinal receptors [33].

We, and others, have reported that tryptophan, given in capsules or infused intraduodenally at a dose of ~3 g, potently reduces energy intake in healthy, normal-weight volunteers [12,17]. Surprisingly, in the current study, appetite ratings and overall energy intake were not affected by tryptophan, however, we observed a substantial reduction in energy intake in ~50% of both the lean and obese participants. In our previous study [12], energy intake in response to an intraduodenal tryptophan infusion (in the absence of other nutrients) was related to concentrations of plasma glucagon and CCK, and strongly related to plasma tryptophan concentrations. The correlations found in the current study support an effect of tryptophan on energy intake, and indicate a role for plasma tryptophan and glucagon concentrations, but not CCK. The presence of other nutrients may have also reduced the potency of tryptophan to diminish energy intake, perhaps by attenuating signals arising from interaction of tryptophan with receptors in the GI tract or by inhibiting its transport across the blood brain barrier, with variable effects across subjects. Furthermore, no relationship between energy intake with plasma tryptophan or glucagon was evident in the obese, also suggesting alterations in energy intake regulation by tryptophan in obese individuals. This may well be related to the smaller increase in plasma tryptophan (~4- vs. 6-fold increase compared to control in obese vs. lean after 3 g of tryptophan) in this group. The reason for this is uncertain, but may potentially reflect enhanced degradation of tryptophan in the liver [42]; thus, higher doses of tryptophan, taking into account differences in body weight, may have resulted in outcomes similar to those in lean subjects.

Some limitations of our study should be noted. We included only male volunteers to exclude effects of the menstrual cycle on energy intake [43]. A meal associated with a greater glycemic response may result in more potent blood glucose-lowering by tryptophan. Blood glucose was assessed using a portable glucometer, which has less than optimal precision. A shorter interval between tryptophan infusion and meal ingestion, or higher doses, potentially calculated per kg body weight, and in the absence of other nutrients, may result in more consistent suppression of energy intake, as well as more potent effects, in obese individuals. However, our doses were based on previous studies showing a potent effect of tryptophan at ~3 g on energy intake in normal-weight volunteers [12,17].

## 5. Conclusions

In conclusion, our study comprehensively investigated the effects of tryptophan on postprandial blood glucose and energy intake in lean and obese volunteers, including underlying GI mechanisms, particularly gastric emptying and gut hormones, and established that tryptophan, administered intragastrically at 3 g, but not 1.5 g, slows gastric emptying of a mixed-nutrient drink, associated with an attenuated glycemic response, but did not consistently reduce energy intake. Further studies to investigate the energy intake-suppressant effects of tryptophan in obese individuals, in the absence of other nutrients, and the relationship with plasma tryptophan concentrations, as well as the blood glucose-regulatory effect of tryptophan in patients with type 2 diabetes, are warranted.

## Figures and Tables

**Table 1:** AUCs_0–60min_ of blood glucose and plasma C-peptide, glucagon, cholecystokinin (CCK) and tryptophan concentrations in response to, and gastric emptying of, a mixed-nutrient drink, ingested 15 min after intragastric infusion of tryptophan, at doses of 1.5 g (‘Trp-1.5g’) or 3.0 g (‘Trp-3.0g’), or control.

	Normal-Weight			Obese		
	Control	Trp-1.5g	Trp-3.0g	Control	Trp-1.5g	Trp3.0 g
Blood glucose (mmol/L*min)	369 ± 14	373 ± 11	360 ± 10	411 ± 10	418 ± 14	397 ± 15
Plasma C-peptide (nmol/L*min)	64 ± 6	63 ± 6	53 ± 8 *^,θ^	129 ± 10	132 ± 11	120 ± 14
Plasma glucagon (pg/mL*min)	3862 ± 268	4021 ± 281	4740 ± 308 ^^,@^	5413 ± 387	5824 ± 408	6382 ± 553 ^δ,θ^
Plasma CCK (pmol/L*min)	249 ± 16	242 ± 20	244 ± 22	260 ± 29	256 ± 28	252 ± 31
Gastric emptying (%recovery of ^13^CO_2_/hour*min)	960 ± 68	1005 ± 46	768 ± 75 ^^,@^	969 ± 47	986 ± 38	832 ± 55 ^π,+^
Plasma tryptophan (µmol/L*min)	4457 ± 138	17,264 ± 1368 ^	27,652 ± 2042 ^^,@^	4643 ± 163	13,442 ± 841 ^	20,816 ± 1914 ^^,@^

Data are means ± SEM, *n* = 16. AUCs_0–60min_ of blood glucose and plasma C-peptide, glucagon, CCK and tryptophan concentrations were analyzed using repeated-measures ANOVA. Differences between treatments were assessed using paired *t*-tests, with adjustments for multiple comparisons made within each outcome using Bonferroni’s correction. Significantly different from control (^ *P* < 0.001; δ *P* < 0.01; * *P* < 0.05) or Trp-1.5g (@ *P* < 0.001; θ *P* < 0.01; π *P* < 0.05). Trend for difference from control (+ *P* = 0.091).

## Supplementary Materials

The following are available online at http://www.mdpi.com/2072-6643/10/4/463/s1, Figure S1: Hunger (A,E), desire to eat (B,F), prospective consumption (C,G) and fullness (D,H) at baseline (*t* = −20 min) and in response to an oral mixed-nutrient drink (*t* = 0 min) following intragastric infusion of tryptophan, at doses of 1.5 g (‘Trp-1.5g’) or 3.0 g (‘Trp-3.0g’), or control, in healthy normalweight (A–D) and obese (E–H) volunteers. Data are means±SEM, *n* = 16, Figure S2: Nausea (A,C) and bloating (B,D) at baseline (*t* = −20 min) and in response to an oral mixed-nutrient drink (*t* = 0 min) following intragastric infusion of tryptophan, at doses of 1.5 g (‘Trp-1.5g’) or 3.0 g (‘Trp-3.0g’), or control, in healthy normal-weight (A,B) and obese (C,D) volunteers. Data are means ± SEM, *n* = 16.
