# Supplementary material for: Effects of Intragastric Administration of Tryptophan on the Blood Glucose Response to a Nutrient Drink and Energy Intake, in Lean and Obese Men
Source: Nutrients. 2018 Apr 8;10(4):463. doi: 10.3390/nu10040463 (PMC5946248; doi:10.3390/nu10040463)
Supplement: Supplementary file 1 [file nutrients-10-00463-s001.pdf]

Supplemental Figure 1

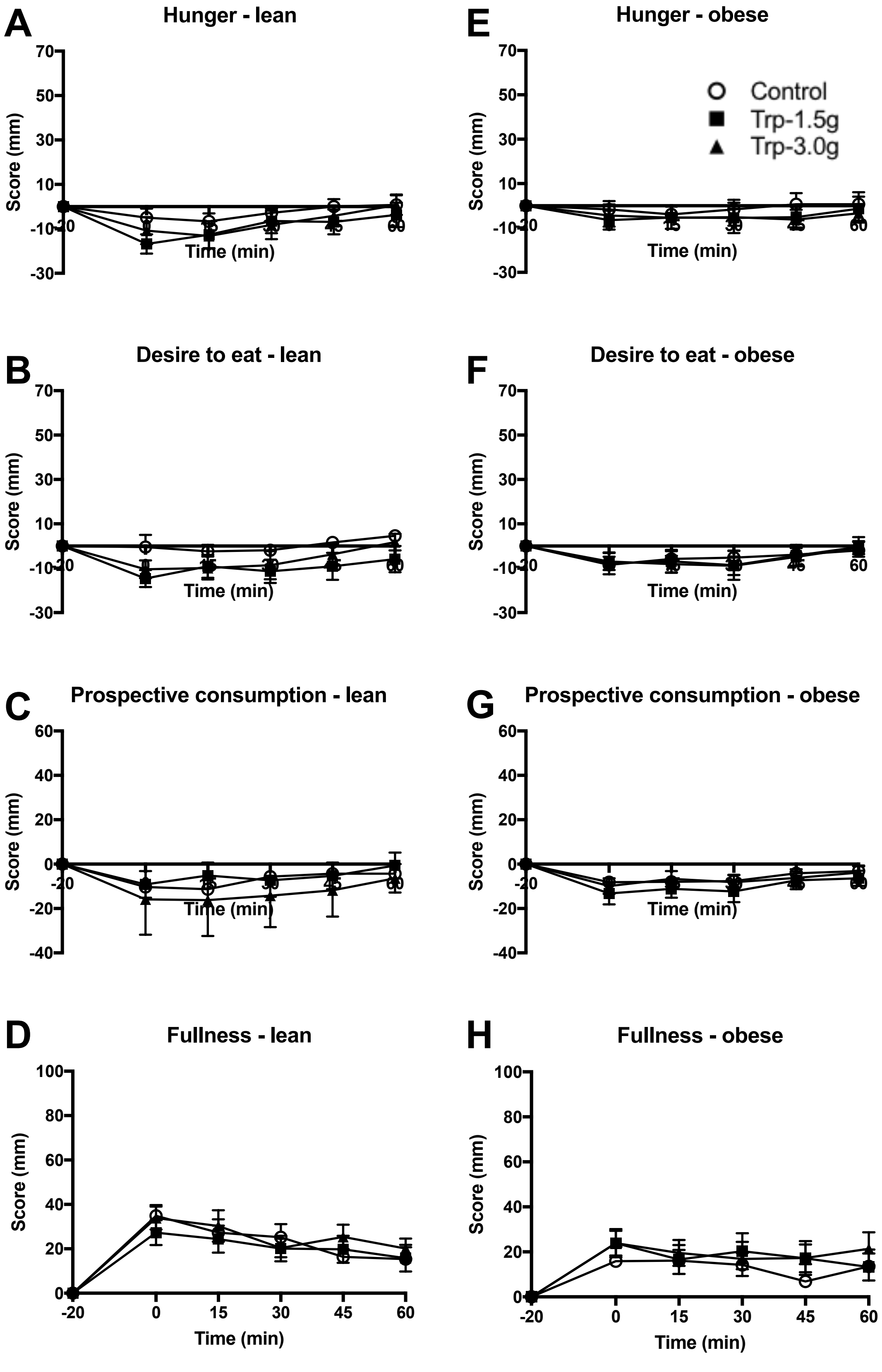

Supplemental Figure 1: Hunger (A, E), desire to eat (B, F), prospective consumption (C, G) and fullness (D, H) at baseline (t=-20 min) and in response to an oral mixed-nutrient drink (t=0 min) following intragastric infusion of tryptophan, at doses of 1.5 g ('Trp-1.5g') or 3.0 g ('Trp-3.0g'), or control, in healthy normal-weight (A-D) and obese (E-H) volunteers. Data are means±SEM, n=16.

Supplemental Figure 2

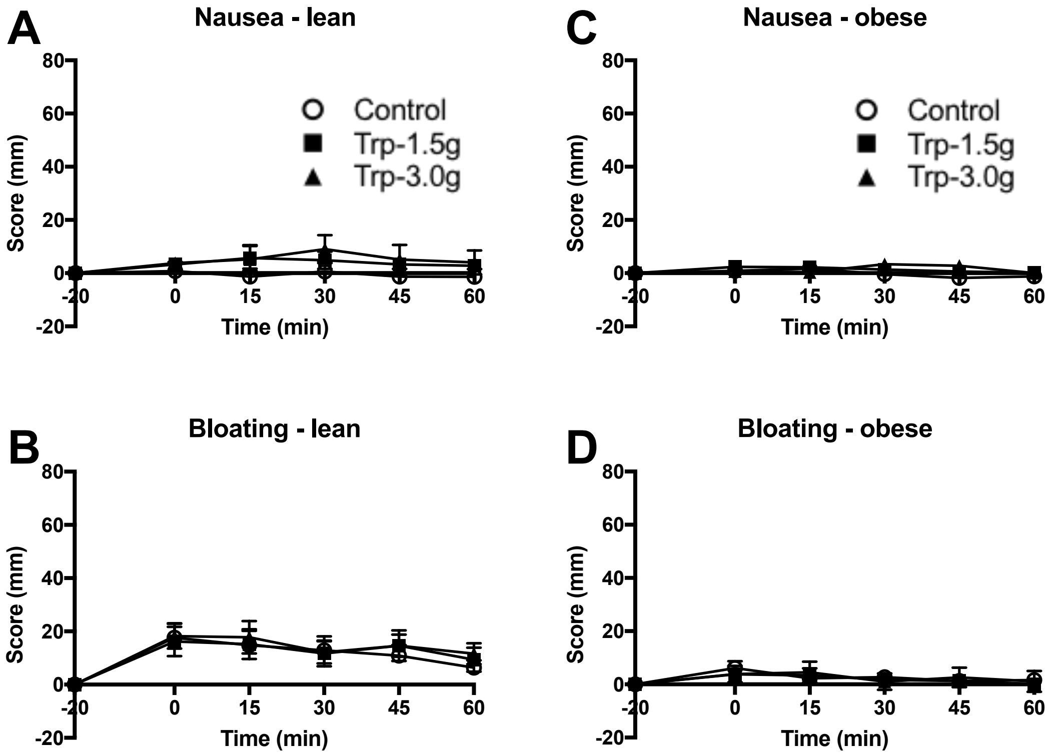

Supplemental Figure 2: Nausea (A, C) and bloating (B, D) at baseline (t=-20 min) and in response to an oral mixed-nutrient drink (t=0 min) following intragastric infusion of tryptophan, at doses of 1.5 g ('Trp-1.5g') or 3.0 g ('Trp-3.0g'), or control, in healthy normal-weight (A, B) and obese (C, D) volunteers. Data are means±SEM, n=16.
